# Global Trends and Forecast of the Burden of Adverse Effects of Medical Treatment: Epidemiological Analysis Based on the Global Burden of Disease Study

**DOI:** 10.7759/cureus.7250

**Published:** 2020-03-12

**Authors:** Moien AB Khan, Elpidoforos S Soteriades, Jeff King, Romona Govender, Muhammad Jawad Hashim, Shamaila Masood-Husain, Syed Fahad Javaid, Shaikha Debaib Mohammed Saeed Al Darei, Shamma Dahi Al Sheryani, Javaid Nauman

**Affiliations:** 1 Family Medicine, College of Medicine and Health Sciences, United Arab Emirates University, Al Ain, ARE; 2 Epidemiology and Public Health, Institute of Public Health, College of Medicine and Health Sciences, United Arab Emirates University, Al Ain, ARE; 3 Family Medicine, United Arab Emirates University, Al Ain, ARE; 4 Family Medicine, Parchmore Medical Centre, London, GBR; 5 Psychiatry and Behavioral Science, College of Medicine and Health Sciences, United Arab Emirates University, Al Ain, ARE; 6 Family Medicine, College of Medicine and Health Sciences, Al Ain, ARE; 7 Family Medicine, Ambulatory Health Services, Al Ain, ARE

**Keywords:** complication of treatment, health policy, health care system, disability, adverse events, patient safety, global burden, yll, yld, daly

## Abstract

Aim

To quantify and update the years of life lost (YLL), years lived with disability (YLD) and disability-adjusted life years (DALY) due to the adverse effects of medical treatment (AEMT) between 1990 and 2017.

Subject and methods

We analyzed the latest dataset from the Global Burden of Disease (GBD) 2017 study. We described the burden of AEMT based on the number of DALY. We additionally evaluated the global age and sex-specific DALY and compared the age-standardized rates of DALY across the World Health Organization (WHO) regions from 1990 to 2017.

Results

Worldwide, the total DALYs due to AEMT were 84.93 [95% uncertainty interval (UI), 62.52 to 102.21] in 1990 and 62.79 (52.09 to 75.45) in 2017 per 100,000 population. The global percentage of change in DALY showed a negative trend of −26.06 % (−41.52 to −10.59) across all WHO regions between 1990 and 2017. The YLD has increased during the period from 1997 to 2017 by 29.47% (17.87 to 41.06). In 2017, men were affected more than women with a DALY of 66.78 in comparison to 58.91 DALY in women. DALY rates per 100,000 were highest across all the WHO regions in the first years of life. The predicted DALY rates were 59.92 (57.52 to 62.32) in the year 2020, 50.36 (32.03 to 68.70) in 2030, and 40.8 (−1.33 to 82.93) in 2040.

Conclusion

Using the GBD 2017 study data, we found a decrease in the DALY rate due to AEMT between 1990 and 2017 with a varying range of DALY between different WHO regions. DALY also differed by age and sex. The forecasting analyses showed a decrease in DALY due to AEMTs with a significant drop in the European region when compared to the African and American regions. However, the increasing trend for YLD signifies an increasing burden of people living with poor health due to AEMT. Our study proposes to identify disability due to AEMT as a significant public health crisis and calls for policymakers to create a robust revised policy.

## Introduction

In 2000, the Institute of Medicine released a groundbreaking report estimating that between 44,000 and 98,000 people die annually in US hospitals because of medical errors [1]. The fact that inappropriate medical care was one of the leading causes of death in a country that is considered to have one of the best health care systems shocked the world, resulting in Congressional hearings and the development of error-reporting systems [2].

The additional attention brought to the serious problem of medical errors led to the collection of data regarding the adverse effects of medical treatment (AEMT), which enabled analyses of common causes, which are often multifactorial and occur in combination. One of the significant contributions of the report by the Institute of Medicine was the finding that the problem was often related to systems of care and that “good people are working in bad systems that need to be safer” [3]. Previously, many medical errors were likely to go undetected because another layer was able to compensate for them before they led to harm. Earlier studies have also reported that the majority of cases of AEMT are preventable, which lent hope that harm could be reduced or perhaps even eliminated [4-5]. Since then, the amount of data collection regarding medical errors has increased dramatically, allowing us to assess not only mortality from medical errors but also the rates of morbidity. Such AEMTs present as both a significant economic and a social burden. Some of those who survive medical errors may still suffer significant harm leading to disability, which, in turn, affects the quality of life and incurs the costs of continuing medical care and lost productivity. AEMT cost the US economy alone an estimated USD 1 trillion in 2008 as a direct health care cost [6]. AEMT is also a substantial burden on society and on the person affected with loss of health that is measured in disability-adjusted life years (DALY) [7]. Thus, evaluating both morbidity and mortality provides a more accurate picture of both the incidence as well as the burden of AEMT [8].

Therefore, the present study aimed to analyze the most recent available global data regarding AEMT and the associated morbidity as measured in DALY. We present the estimates related to DALY and forecast possible emerging trends segregated by the World Health Organization (WHO) regions and selected countries using the Global Burden of Diseases, Injuries and Risk Factor Study (GBD 2017) [9].

## Materials and methods

The GBD 2017 is an active global collaboration of more than 1,800 researchers across 195 countries. GBD study research measures and quantifies the degree of health loss from disease, injuries, and risk factors by age, sex, and global locations through specific points in time [10]. The Institute of Health Metrics and Evaluation at the University of Washington, Seattle, manages the GBD repository [9].

The GBD study uses the International Classification of Diseases (ICD) codes (ICD-9 and ICD-10) to extract data related to AEMT from the data sources. A detailed list of the ICD-9 and ICD-10 codes used for AEMT in health care settings and hospital claims analysis is available in the supplementary appendix of the GBD paper [11]. For instance, ICD-10 codes T36 - T50 were used for poisoning by adverse effects of the underdosing of drugs, medicaments, and biological substances. These AEMT data are obtained from multiple sources such as inpatient, outpatient, and emergency care records, as well as records from health insurance claims. The data are later pooled through various sophisticated mathematical modeling and statistical estimates by age, cause, country, sex, and year. A detailed description of the methodology of the GBD study is provided elsewhere [12-13].

Data sources

We retrieved and analyzed the epidemiological data attributed to AEMT from the latest GBD 2017 dataset and quantified the results by the WHO regional groups [9]. Age-standardized rates were used to compensate for the variation in different age groups. For our study, we extracted age-standardized YLL, YLD, and DALY with 95% uncertainty intervals using the GBD interactive data visualization and the GBD Compare tool. The GBD Compare is an interactive tool to analyze updated estimates of the world's health for 359 diseases and injuries and 84 risk factors from 1990 to 2017.

WHO regional groups

The data from the GBD 2017 study were extracted based on the WHO regions of Africa, the Americas, Southeast Asia, Europe, the Eastern Mediterranean region, and the Western Pacific region (https://www.who.int/chp/about/regions/en). We also selected countries within the WHO regions based on the population and the continents for useful comparison. The WHO administrative regions are grouped together to address health care and social issues. Countries within the region collaborate to improve health within the area for health development. Such organizational collaboration also helps provide policies for health promotion and the prevention of disease and injury by working with intergovernmental bodies and other nongovernmental organizations (NGOs).

Uncertainty intervals

The data that is added for modeling to estimate the burden of disease can have varying degrees of uncertainty or errors. Uncertainty interval (UI) refers to a widely used confidence interval and represents a range of values that are likely to include the correct estimate of health loss for a given cause where limited data create substantial uncertainty. The UI is generated by sampling the values across age, cause, and location and is represented as 2.5 and 97.5-centile values as lower bound and upper bound values, respectively, and provide a correct estimate of health loss due to AEMT [12,14]. The UIs generally include all sources of uncertainty, including those arising from systematic biases and measurement errors. In contrast, generally reported confidence intervals are based solely on the variation observed in sample data.

Trends and percentage change

The percentage difference between the two values defines the pattern or degree of change. The percentage change could increase, decrease or stay the same, and in the present study, we calculated the percentage change between the years 1990 and 2017.

Years of life lost (YLL) and years lived with disability (YLD)

YLL and YLD are the two constituents of DALY. YLL contributes to the mortality aspect of DALY and is the difference between standard life expectancy and age at death. YLD accounts for the morbidity part of DALY and indicates the number of years of life lived in less than full health [15].

Disability-adjusted life years (DALY)

DALY is the sum of YLL and YLD. One DALY equals one year of healthy life lost. DALY measures and estimates the overall disease burden by cumulating the total health loss at a population level as a single variable. Calculating DALY enables estimations of the total number of years lost due to AEMT at the national and regional levels. Thus, it is a more accurate measure of human suffering resulting from AEMT than mortality or incidence alone [9].

Statistical data analysis

We calculated the age and sex-specific percentage differences of GBD estimates of YLL, YLD, and DALY between the years 1990 and 2017 for every WHO region. The Statistical Package for the Social Sciences (SPSS Statistics for Windows, version 23.0, Chicago, IL) was used to conduct statistical analyses. SPSS Time-series Modeler with the expert modeler option without any events was used to forecast the DALY until 2040. With the forecasting, none of the observed values were marked as outliers.

## Results

YLL, YLD, and DALY

Table 1 shows the worldwide age-standardized YLL, YLD, and DALY rates per 100,000 with 95% UIs due to AEMT. The estimates of YLL, YLD, and DALY varied between countries and regions. In 2017, the highest YLL were estimated in the African region [100.09 (95% UI, 84.47 to 136.14)] while the lowest was seen in the Western Pacific region [23.7 (21.87 to 28.11)]. The highest YLD was estimated in the region of the Americas [11.84 (7.38 to 17.72)], while the lowest was in the African region [1.84 (1.14 to 2.78)] in 2017. Similarly, across the selected countries, the US [26.78 (16.72 to 39.96)] recorded the highest YLD while the lowest was in Brazil [0.96 (0.59 to 1.45)] (Table 1).

**Table 1:** Estimates of disability-adjusted life years due to the adverse effects of medical treatment by World Health Organizational regions, 1990-2017 YLL: Years of Life Lost, YLD: Years Lived with Disability, DALY: Disability Adjusted Life Years, UI: Uncertainty Intervals The estimates are age-standardized per 100,000 population.

Region	YLL Values (95% UI)		YLD Values (95% UI)		DALY Values (95% UI)	
	1990	2017	1990	2017	1990	2017
Global	81.78 (59.33 - 99)	58.33 (48.14 - 70.92)	3.14 (1.95 - 4.72)	4.45 (2.79 - 6.68)	84.93 (62.52 - 102.21)	62.79 (52.09 - 75.45)
African Region	136.12 (94.85 - 207.52)	100.09 (84.47 - 136.14)	1.72 (1.06 - 2.58)	1.84 (1.14 - 2.78)	137.85 (96.61 - 209.91)	101.93 (85.97 - 138.08)
Angola	141.9 (81.69 - 253.66)	79.14 (61.27 - 120.04)	1.7 (1.05 - 2.54)	1.78 (1.09 - 2.71)	143.6 (83.79 - 255.31)	80.92 (62.78 - 121.65)
Ghana	208.11 (170.45 - 258.05)	198.82 (147.48 - 252.11)	2.52 (1.59 - 3.79)	2.81 (1.73 - 4.21)	210.64 (173.35 - 260.26)	201.63 (149.79 - 255.2)
Kenya	78.9 (61.78 - 116.2)	61.34 (48.06 - 87.83)	1.36 (0.84 - 2.05)	1.5 (0.92 - 2.28)	80.26 (63.11 - 117.28)	62.84 (49.44 - 89.63)
South Africa	72.29 (53.19 - 85.09)	47.14 (37.52 - 63.44)	1.8 (1.11 - 2.7)	1.92 (1.18 - 2.91)	74.09 (54.87 - 86.71)	49.06 (39.56 - 65.54)
Eastern Mediterranean Region	109.33 (77.64 - 129.14)	83.17 (66.28 - 94.33)	2.08 (1.28 - 3.12)	2.39 (1.48 - 3.58)	111.41 (79.82 - 131.3)	85.56 (68.25 - 96.3)
Egypt	68.74 (40.7 - 97.36)	38.88 (24.29 - 55.03)	2.03 (1.26 - 3.05)	2.43 (1.49 - 3.67)	70.77 (42.83 - 99.46)	41.31 (26.79 - 57.43)
Saudi Arabia	155.54 (100.13 - 207.45)	128.4 (76.96 - 180.7)	4.13 (2.58 - 6.12)	4.07 (2.53 - 6.09)	159.68 (104.26 - 211)	132.48 (81.63 - 184.99)
European Region	44.11 (36.37 - 48.56)	30.96 (28.6 - 37.68)	2.91 (1.81 - 4.36)	3.28 (2.04 - 4.95)	47.02 (39.26 - 51.81)	34.24 (31.33 - 41.08)
Germany	26.28 (22.95 - 29.97)	28.41 (21.67 - 32.13)	3.79 (2.35 - 5.77)	4.27 (2.64 - 6.51)	30.08 (26.54 - 33.7)	32.68 (25.73 - 37.13)
Kazakhstan	55.27 (44.37 - 63.12)	45.31 (39.88 - 63.36)	2.47 (1.53 - 3.66)	2.88 (1.77 - 4.33)	57.75 (46.66 - 65.27)	48.2 (42.43 - 65.93)
Poland	37.05 (28.49 - 39.75)	29.32 (26.02 - 36.79)	2.57 (1.6 - 3.86)	4.01 (2.5 - 5.96)	39.62 (30.92 - 42.68)	33.33 (29.58 - 40.71)
Russian Federation	38.96 (36.26 - 53.18)	41.99 (36.85 - 51.06)	1.77 (1.11 - 2.67)	2.2 (1.38 - 3.31)	40.74 (37.83 - 55.24)	44.2 (39 - 53.29)
Sweden	14.12 (13.07 - 18.96)	14.81 (12.58 - 17.08)	4.07 (2.52 - 6.24)	6.02 (3.68 - 9.14)	18.2 (16.02 - 22.97)	20.84 (17.82 - 24.7)
Ukraine	23.8 (19.78 - 27.58)	37.3 (32.42 - 56.52)	1.36 (0.85 - 2.05)	1.81 (1.12 - 2.75)	25.17 (21.08 - 28.81)	39.12 (34.21 - 58.24)
United Kingdom	20.21 (19.39 - 24.93)	23.93 (18.08 - 25.07)	3.03 (1.85 - 4.69)	2.19 (1.37 - 3.3)	23.25 (21.54 - 28.16)	26.12 (20.19 - 28.01)
Region of the Americas	73.22 (59.94 - 78.26)	43.46 (40.81 - 56.95)	7.77 (4.87 - 11.68)	11.84 (7.38 - 17.72)	81 (67.96 - 88.06)	55.3 (49.12 - 69.82)
Argentina	134.82 (100.11 - 144.27)	84.24 (73.51 - 109.98)	4.97 (3.12 - 7.38)	6.82 (4.24 - 10.15)	139.8 (104.33 - 149.59)	91.06 (80 - 116.12)
Brazil	80.7 (62.07 - 86.34)	50.93 (47.43 - 67.4)	0.79 (0.49 - 1.19)	0.96 (0.59 - 1.45)	81.49 (62.94 - 87.1)	51.89 (48.42 - 68.51)
Cuba	72.97 (44.32 - 79.51)	19.54 (15.61 - 37.49)	1.22 (0.75 - 1.82)	1.62 (1.01 - 2.51)	74.19 (45.56 - 80.71)	21.16 (17.12 - 39.19)
Mexico	69.3 (60.47 - 74.99)	41.89 (39.09 - 56.96)	0.99 (0.62 - 1.49)	1.16 (0.71 - 1.74)	70.3 (61.43 - 76.03)	43.05 (40.16 - 58.19)
United States	33.89 (30.49 - 39.31)	27.86 (26.25 - 35.1)	15.72 (9.84 - 23.69)	26.78 (16.72 - 39.96)	49.61 (42.93 - 58.38)	54.65 (44.04 - 68.43)
Southeast Asian Region	110.34 (76.14 - 132.9)	25.02 (23.03 - 29.84)	1.28 (0.79 - 1.92)	4.52 (2.83 - 6.77)	111.62 (77.27 - 134.08)	29.55 (26.49 - 34.59)
China	67.07 (42.87 - 79.68)	17.88 (16.03 - 22.85)	3.14 (1.95 - 4.74)	5.16 (3.21 - 7.78)	70.22 (46.17 - 83.17)	23.04 (19.91 - 28.4)
India	118.59 (77.5 - 146.79)	96.35 (67.15 - 112.02)	1.23 (0.76 - 1.84)	1.7 (1.05 - 2.57)	119.83 (78.75 - 147.81)	98.05 (68.97 - 113.74)
Indonesia	76.48 (57.14 - 100.32)	53.12 (42.42 - 63.59)	1.33 (0.82 - 2.03)	1.09 (0.65 - 1.69)	77.81 (58.54 - 102.11)	54.21 (43.37 - 64.78)
Western Pacific Region	61.44 (41.8 - 71.49)	23.7 (21.87 - 28.11)	2.8 (1.74 - 4.22)	4.6 (2.88 - 6.9)	64.25 (44.61 - 74.76)	28.31 (25.36 - 32.96)
Australia	23.63 (20.35 - 26.39)	20.11 (16.72 - 23.06)	10.84 (6.74 - 16.24)	22.64 (14.16 - 33.74)	34.47 (29.64 - 40.47)	42.76 (33.97 - 54.28)
Fiji	90.34 (71.77 - 107.18)	81.32 (65.09 - 97.28)	2.81 (1.74 - 4.2)	3.39 (2.09 - 5.08)	93.15 (74.52 - 110.64)	84.71 (68.47 - 100.77)
Japan	12.21 (11.52 - 15.64)	14.1 (10.74 - 15.3)	1.18 (0.71 - 1.81)	1.15 (0.71 - 1.73)	13.39 (12.45 - 16.81)	15.26 (11.85 - 16.71)

In 2017, the total global DALY due to AEMT was 62.79 (52.09 to 75.45) per 100,000 population. DALY rates across WHO regions in 2017 varied from a low [28.31 (25.36 to 32.96)] in the Western Pacific region to a high [101.93 (85.97 to 138.08)] in the African region. Among the selected countries, the highest DALY due to AEMT in 2017 was in Ghana [201.63 (149.79 to 255.2)] and the lowest was recorded in Japan [15.26 (11.85 to 16.71)].

Worldwide, the percentage change in DALY showed an overall negative trend [−26.06 % (−41.52 to −10.59)] between 1990 and 2017 (Table 2). The estimated DALY changes were −73.52% (−91.25 to −55.78)] in the Southeast Asian region, −23.2% (−38.7 to −7.69) in the Eastern Mediterranean region, and −55.92% (−73.88 to −37.95) in the Western Pacific region (Table 2).

**Table 2:** Percentage change between 1990 and 2017 in disability-adjusted life years due to the adverse effects of medical treatment by World Health Organizational regions YLL: Years of Life Lost, YLD: Years Lived with Disability, DALY: Disability Adjusted Life Years, UI: Uncertainty Intervals The estimates are age-standardized per 100,000 population.

	Percentage Change (95% UI)	Percentage Change (95% UI)	Percentage Change (95% UI)
Region	YLL	YLD	DALY
Global	-28.67 (-44.44 to -12.89)	29.47 (17.87 to 41.06)	-26.06 (-41.52 to -10.59)
African Region	-26.47 (-40.71 to -12.22)	6.36 (-4.3 to 17.02)	-26.05 (-40.77 to -11.32)
Angola	-44.22 (-58.52 to -29.91)	4.62 (-5.97 to 15.21)	-43.64 (-58.38 to -28.89)
Ghana	-4.46 (-19.07 to 10.15)	10.26 (-0.44 to 20.96)	-4.27 (-19.14 to 10.6)
Kenya	-22.25 (-37.25 to -7.24)	9.41 (-1.43 to 20.25)	-21.7 (-36.96 to -6.43)
South Africa	-34.79 (-49.8 to -19.77)	5.99 (-4.84 to 16.82)	-33.78 (-49.05 to -18.5)
Eastern Mediterranean Region	-23.92 (-39.19 to -8.64)	13 (2.05 to 23.94)	-23.2 (-38.7 to -7.69)
Egypt	-43.42 (-59 to -27.83)	16.07 (4.92 to 27.21)	-41.62 (-57.44 to -25.79)
Saudi Arabia	-17.44 (-33.21 to -1.66)	-1.5 (-12.86 to 9.86)	-17.03 (-33 to -1.05)
European Region	-29.82 (-45.94 to -13.69)	11.44 (0.08 to 22.79)	-27.17 (-43.5 to -10.83)
Germany	8.11 (-8.37 to 24.59)	11.08 (-0.47 to 22.63)	8.66 (-8.02 to 25.34)
Kazakhstan	-18.02 (-34.55 to -1.48)	14.3 (2.54 to 26.05)	-16.53 (-33.37 to 0.31)
Poland	-20.87 (-37.81 to -3.92)	35.87 (23.85 to 47.88)	-15.88 (-33.16 to 1.4)
Russian Federation	7.79 (-9.61 to 25.19)	19.29 (7.02 to 31.55)	8.49 (-9.26 to 26.24)
Sweden	4.89 (-12.58 to 22.36)	32.31 (19.7 to 44.91)	14.49 (-3.46 to 32.44)
Ukraine	56.71 (39.13 to 74.28)	24.74 (11.79 to 37.68)	55.41 (37.44 to 73.37)
United Kingdom	18.39 (4.06 to 32.71)	-38.59 (-51.96 to -25.21)	12.34 (-0.28 to 24.96)
Region of the Americas	-40.65 (-54.81 to -26.48)	34.33 (21.72 to 46.93)	-31.72 (-46.48 to -16.95)
Argentina	-37.51 (-50.55 to -24.46)	27.04 (16.1 to 37.97)	-34.86 (-47.71 to -22)
Brazil	-36.88 (-50.42 to -23.33)	17.27 (5.91 to 28.62)	-36.31 (-50.56 to -22.05)
Cuba	-73.22 (-87.31 to -59.12)	24.86 (13.14 to 36.57)	-71.47 (-85.94 to -56.99)
Mexico	-39.55 (-53.09 to -26)	13.97 (1.74 to 26.19)	-38.76 (-53.34 to -24.17)
United States	-17.79 (-32.65 to -2.92)	41.3 (28.03 to 54.56)	10.14 (-5.92 to 26.2)
Southeast Asian Region	-77.31 (-91.92 to -62.69)	71.55 (62.34 to 80.75)	-73.52 (-91.25 to -55.78)
China	-73.34 (-88.89 to -57.78)	39.1 (25.21 to 52.98)	-67.18 (-82.93 to -51.42)
India	-18.75 (-32.99 to -4.5)	27.27 (18.92 to 35.61)	-18.17 (-34.02 to -2.31)
Indonesia	-30.54 (-44.84 to -16.23)	-21.89 (-30.24 to -13.53)	-30.32 (-47.58 to -13.05)
Western Pacific Region	-61.41 (-76.99 to -45.82)	39.04 (27.59 to 50.48)	-55.92 (-73.88 to -37.95)
Australia	-14.88 (-29.88 to 0.12)	52.11 (41.28 to 62.93)	24.02 (6.24 to 41.79)
Fiji	-9.98 (-24.99 to 5.03)	17.18 (5.86 to 28.49)	-9.05 (-27.22 to 9.12)
Japan	15.48 (0.2 to 30.75)	-2.27 (-13.48 to 8.94)	13.91 (-4.58 to 32.4)

**Figure 1:**
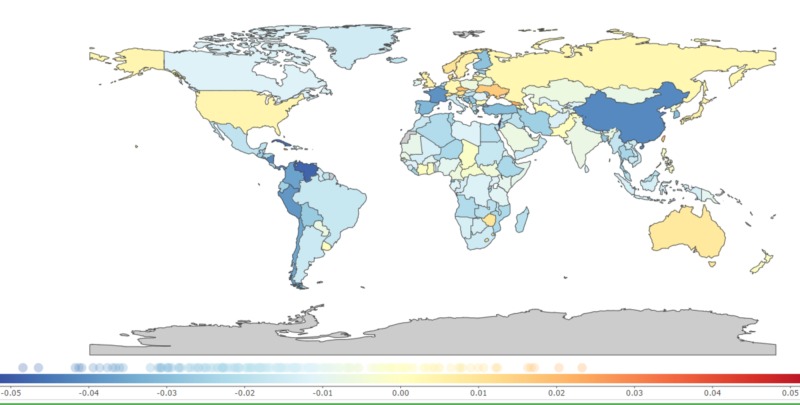
Annual percentage change in age-standardized disability-adjusted life years due to the adverse effects of medical treatment, 1990-2017 0.01 on the color bar graph indicates a 1% annual increase of disability-adjusted life years per 100,000 population from 1990 to 2017 and -0.01 indicates a -1% annual decrease of disability-adjusted life years per 100,000 population from 1990 to 2017 [9].

The countries with the most considerable percentage changes in DALY were Cuba [−71.47% (−85.94 to −56.99)], China [−67.18% (−82.93 to −51.42), and Egypt [−41.62% (−57.44 to −25.79)]. However, countries across the different regions, such as Ukraine [55.41% (37.44 to 73.37), Australia [24.02% (6.24 to 41.79), Sweden [14.49% (−3.46 to 32.44)], Japan [13.91% (−4.58 to 32.4)], United Kingdom [12.34% (−0.28 to 24.96)], and the US [10.14% (−5.92 to 26.2)] demonstrated an increasing trend in DALY between 1990 and 2017 (Table 2 and Figure 1).

**Figure 2:**
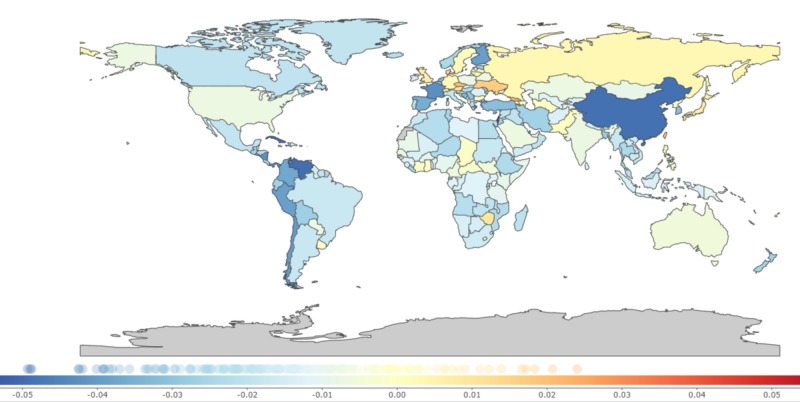
Annual percentage change in age-standardized years of life lost due to the adverse effects of medical treatment, 1990-2017 0.01 on the color bar graph indicates a 1% annual increase of years of life lost per 100,000 population from 1990 to 2017 and -0.01 indicates a -1% annual decrease of years of life lost per 100,000 population from 1990 to 2017 [9].

A significant change in DALY occurred due to YLL (Table 2). Across the countries, the most notable recorded change in YLL was in China [−73.34% (−88.89 to −57.78)] while countries like Ukraine [56.71% (39.13 to 74.28)], Japan [15.48% (0.2 to 30.75)], and United Kingdom [18.39% (4.06 to 32.71)] showed increases in percentage change of YLL. Within the African region, there was a decrease in YLL [−26.47 % (−40.71 to −12.22)], which contributed to a reduction of the DALY. Surprisingly, the African region performed better than the Eastern Mediterranean region [−23.92 % (−39.19 to −8.64)] (Table 2 and Figure 2).

**Figure 3:**
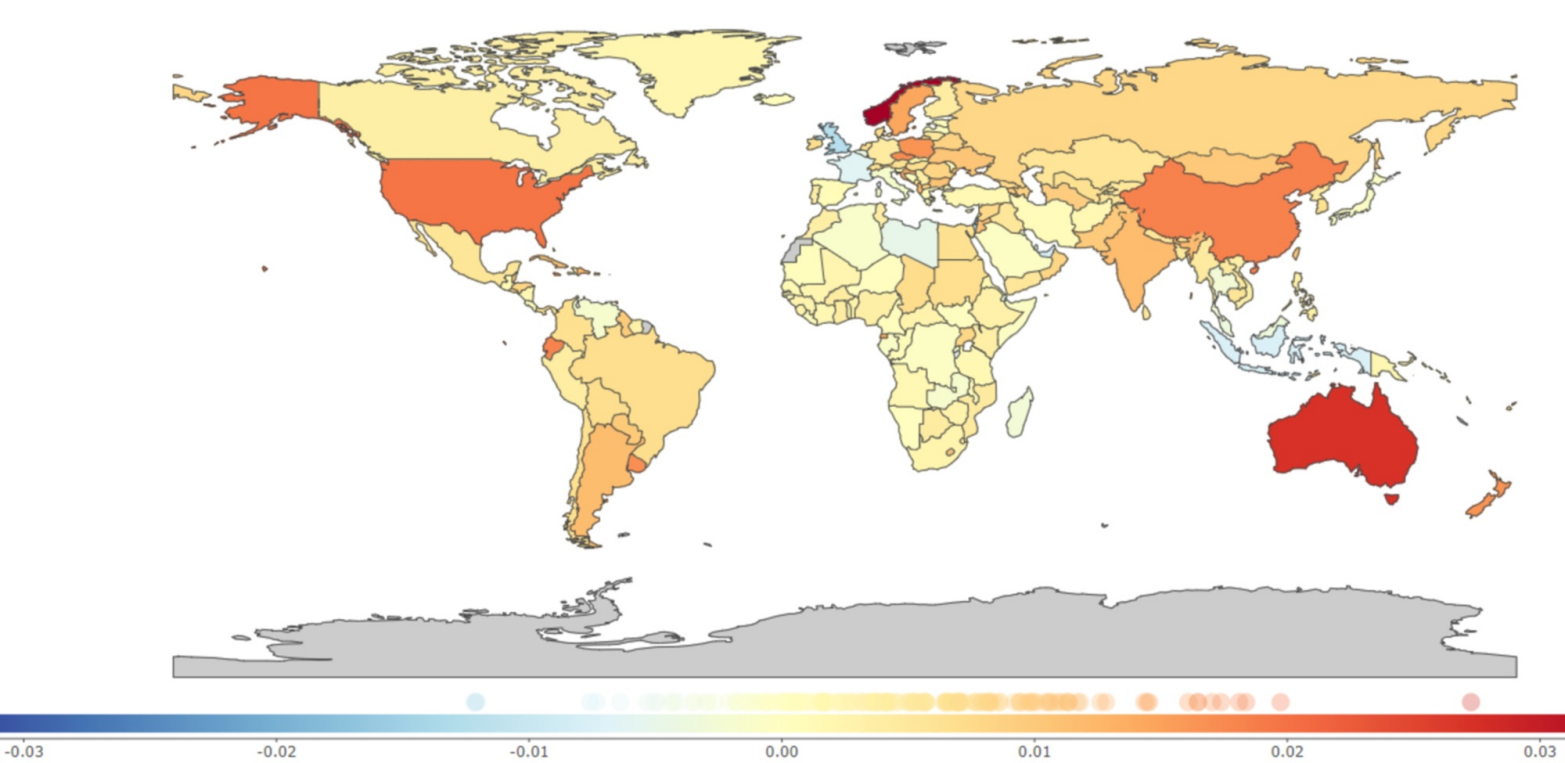
Annual percentage change in age-standardized years lived with disability due to the adverse effects of medical treatment, 1990-2017 0.01 on the color bar graph indicates a 1% percent annual increase of years lived with disability per 100,000 population from 1990 to 2017 and -0.01 indicates a -1% percent annual decrease of years lived with disability per 100,000 population from 1990 to 2017 [9].

The change in YLD rates has been smaller when compared to the percentage change in YLL. Interestingly, YLD increased worldwide by 29.47% (17.87 to 41.06) with the highest increase recorded in the Southeast Asian region [71.55% (62.34 to 80.75)] and the lowest percentage change was among the African region [6.36% (−4.3 to 17.02)]. Australia had the highest YLD [52.11% (41.28 to 62.93)] while the lowest was recorded in the United Kingdom [−38.59% (−51.96 to −25.21)]. Most affluent countries like the United States [41.3% (28.03 to 54.56)], Sweden [32.31% (19.7 to 44.91)], and Germany [11.08% (−0.47 to 22.63)] continue to show increasing trends in YLD while Indonesia [−21.89 % (−30.24 to −13.53) and the United Kingdom [−38.59% (−51.96 to −25.21)] showed a decline. (Table 2 and Figure 3).

DALY by age and sex

**Figure 4:**
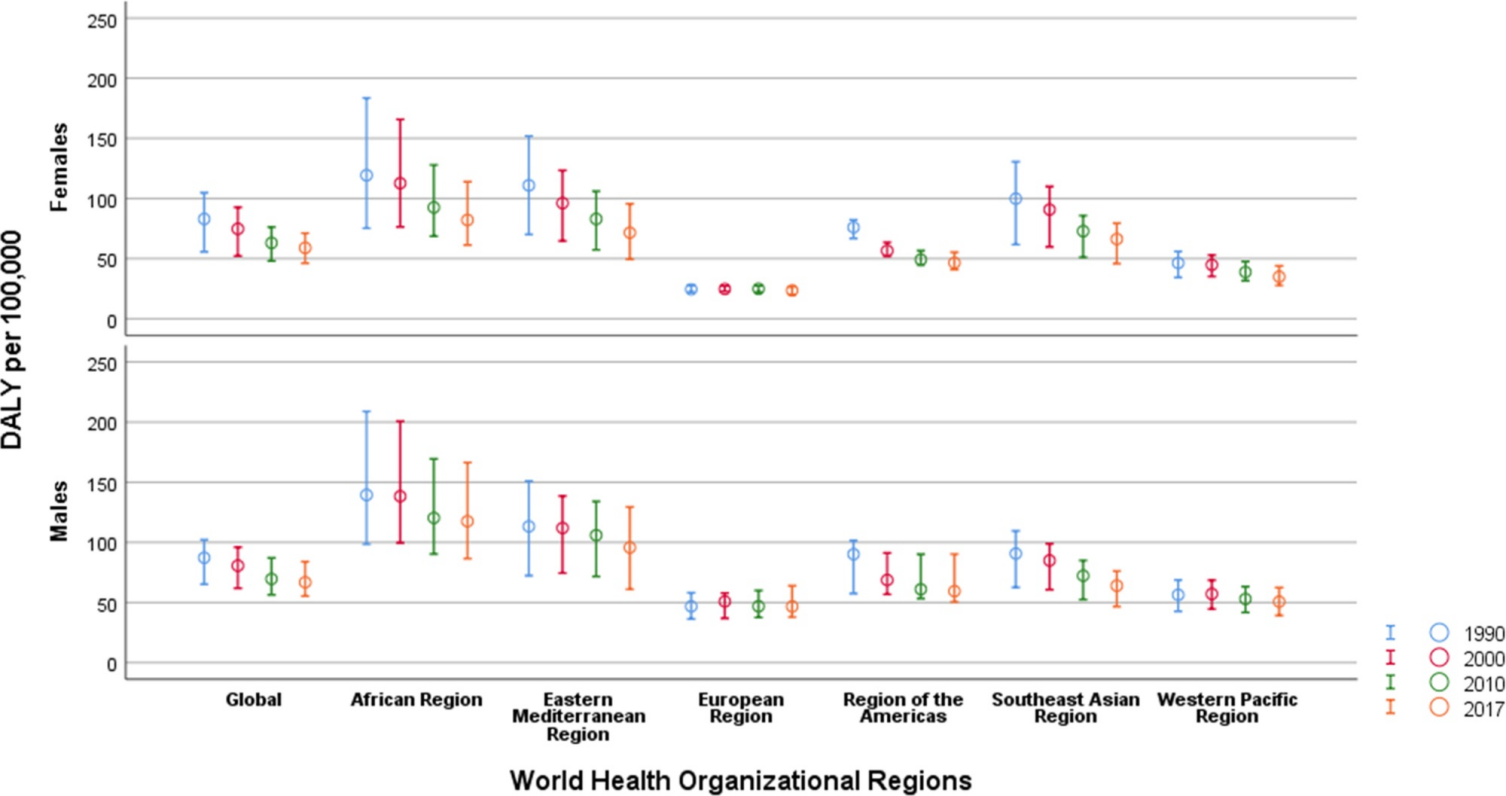
Sex stratified trends in disability-adjusted life years due to the adverse effects of medical treatment by World Health Organizational regions, 1990-2017 DALY: Disability Adjusted Life Years The estimates are age-standardized per 100,000 population.

Figure 4 shows trends in DALY by sex across WHO regions. In 2017, the global estimates for DALY were higher in men [66.78 (55.37 to 84.03)] as compared with women [58.91 (46.17 to 71.12)]. Men had low estimates of DALY in the Southeast Asian region [77.96 (55.32 to 88.37)] and the Eastern Mediterranean region [82.74 (61.84 to 95.30)] (Figure 4). In contrast, a male-dominant DALY rate of [44.35 (39.57 to 58.34)] in comparison to female [24.57 (22.39 to 27.08)] was observed in the European region.

**Figure 5:**
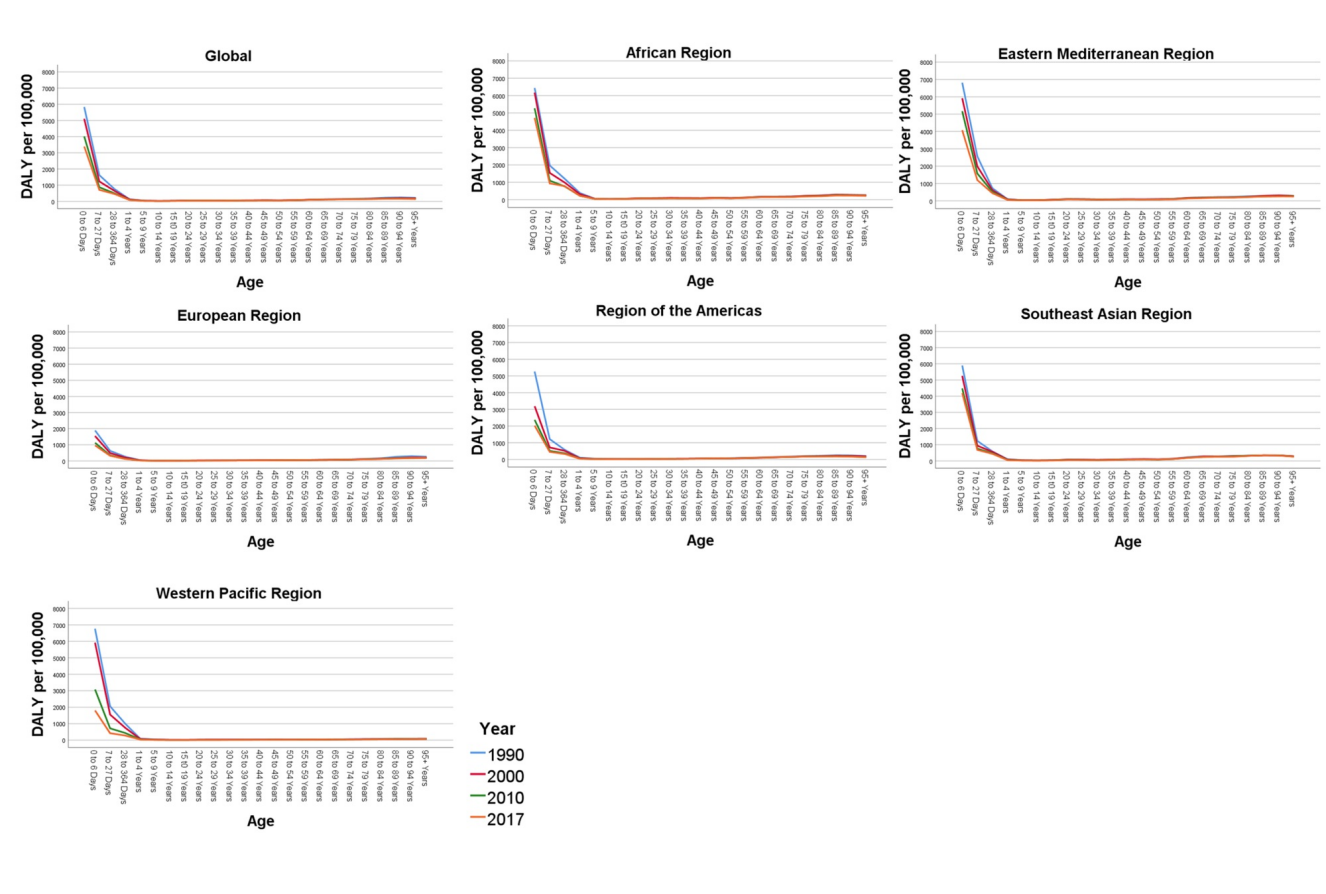
Age-specific trends in disability-adjusted life years due to adverse effects of medical treatment by World Health Organizational regions, 1990-2017 The figure panes depict the overall disability-adjusted life year rates per 100,000 population globally and across the six World Health Organizational (WHO) regions (African region, Eastern Mediterranean region, European Region, Region of the Americas, Southeast Asian Region, and the Western Pacific Region) during the year 1990, 2000, 2010, and 2017.

Figure 5 shows the DALY rate by different age groups in the world regions between 1990 and 2017. DALY rates per 100,000 were highest across all WHO regions in the first year of life, particularly in the newborn period. The highest DALY was observed in the zero to six days age group across the world [3391.0 (2595.31 to 4124.26)]. The results suggest a five-fold rate in the African region [4717.11 (3591.35 to 6842.48)] in comparison to the European region [959.90 (774.83 to 1258.75)].

Between 1990 and 2017, the majority of the contribution to the DALY rates was recorded as being from the 0-1 year groups across all regions. In the age group of 60-64 years, the number of DALY was lowest in the Western Pacific region [35.56 (27.80 to 46.73)] when compared to the highest in the Southeast Asian region [190.15 (136.47 to 222.30)]. With the elderly age group of 70-74 years, patterns of DALY rates were yet again highest in the Southeast Asian region [257.81 (186.47 to 304.53)] compared to the European regions [83.12 (75.15 to 101.22)].

DALY forecasted trends

**Figure 6:**
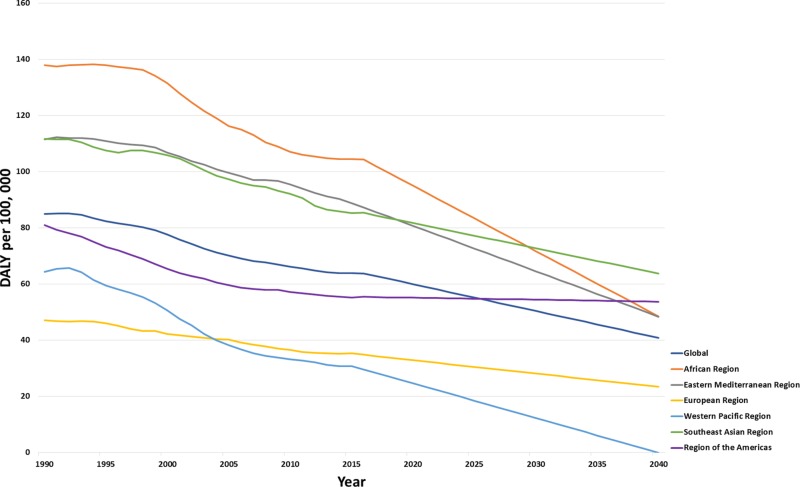
Forecasted trends for age-standardized disability-adjusted life years due to the adverse effects of medical treatment across World Health Organizational regions DALY: Disability-Adjusted Life Years

Globally, the DALY rates are forecasted to decrease from 59.92 (57.52 to 62.32) in 2020, 50.36 (32.03 to 68.7) in 2030, and 40.8 (−1.33 to 82.93) in 2040 (Figure 6). The greatest reduction in DALY is forecasted to be in the Western Pacific region [24.61 (19.44 to 29.78)] as compared to the highest in the African region [94.96 (89.36 to 100.55)] in 2020. While by 2040, the Eastern Mediterranean region [48.33 (−4.2 to 100.86)] will have twice the DALY rates when compared to the European region [23.36 (19.76 to 26.96)]. In the region of the Americas, the predicted DALYs are 55.1 (52.73 to 57.46) in 2020, 54.4 (36.68 to 72.11) in 2030, and 53.7 (13.12 to 94.28) in 2040. On the other hand, a drastic decline in DALY rates is expected within the African region: 94.96 (89.36 to 100.55) in 2020, 71.7 (28.91 to 114.5) in 2030, and 48.45 (9.89 to 146.79) by 2040. In addition, the DALYs are predicted to decrease in the Southeast Asia region: 81.66 (76.33 to 87) in 2020, 72.68 (33.38 to 111.99) in 2030, and 53.7 (13.12 to 94.28) by 2040 (Figure 6).

## Discussion

In this current study, we describe the levels, patterns, and trends of DALY due to AEMT by WHO administrative regions and by selected countries between 1990 and 2017. The main findings of the study are that the age-standardized rates of DALY have decreased significantly since 1990 with a particular reduction in YLL. However, YLD has increased across all WHO regions. The decline in YLL indicates the improvement in clinical outcomes and progress in the reduction of mortality due to AEMT. However, the morbidity component of DALY, i.e., YLD has increased, indicating that more people are living with disability and remain a challenge across all WHO regions. In addition, age-specific DALY has been relatively stable and has varied only minimally by region and sex.

Globally and across WHO regions, we observed that the highest rates of DALY are in the first year of life. The findings of our study suggest that the development of age-specific guidelines and appropriate training to treat children under one year of age are urgently warranted to make medical care intervention safer during this vulnerable period of life [16-17].

At the other extreme of age, patients 65 years and older are more likely to have multiple comorbidities, which makes treatment plans for these patients complex. Multimorbidity is one of the main challenges that health care systems face worldwide, which places a significant burden on health care systems [18]. Current single-system guidelines do not usually take into account multimorbidity and following multiple guidelines for individual conditions increases the risk of polypharmacy and hence the risk of an AEMT.

Among the WHO regions, the European region recorded lower DALY rates in the age group of 65 years and above while the Southeast Asian region had higher rates of DALY across the same age group. These results are comparable with other studies where there is higher morbidity among older patients requiring treatment of chronic diseases, which are particularly challenging in lower-income regions [19-20]. Although the African region had a lesser increase in YLD when compared to the other areas, these results should be viewed with caution, as these regions have lower health care budgets per capita as well as higher burdens of diseases that require complex treatment regimens [21]. Interestingly, China recorded a greater reduction in DALY; however, the decrease in DALY was much lower than the regional average. This indicated a phase of improved clinical care leading to a decrease in mortality, but with the complication of AEMT resulting in the increasingly persistent burden of patients living with a disability-related to an AEMT. Moreover, the DALY rate in India is much higher than the Southeast Asian regional average. Such trends highlight the challenges that economically developing countries like China and India experience with rapidly increasing economic and epidemiological transitions. Nevertheless, AEMTs are underreported in these countries, and the YLL, YLD and DALY estimates may not represent the full impact of the future burden due to AEMTs [22].

Globally, men, in general, had a higher burden of DALY, potentially indicating a higher economic burden for families where the primary wage earner is male. It is estimated that almost three-fourths of the AEMT leads to some form of disability lasting six months or less [23]. Our study suggests that the burden of AEMT indicated by YLD will continue to rise unless new strategies or drastic measures are taken as per the needs across each region and individual countries for the prevention of AEMT. This especially holds true in regions from the lower and middle-income groups, which are growing economically. While the global trends show an increase, the percentage change in YLD has reduced in the United Kingdom, Indonesia, Japan, and Saudi Arabia, which suggests the benefit of studying the strategies implemented in these settings to determine which are beneficial and could be generalized elsewhere.

Although DALY and mortality rates have decreased due to improvements in health care delivery globally, systematizing such achievements will require a continued focus on identifying, developing, and implementing evidence-based processes to deliver safe and effective care. Improving care for patients with multimorbidities will benefit them as well as health care professionals. The development of tools and guidelines that incorporate multimorbidity, an emphasis on proactive and preventative management, and the training of professionals in multimorbidity would assist in the safer management of patients with multiple conditions [24]. A better understanding of the relationship between the growing prevalence of multimorbidity on death and disability as measured by DALY, YLL, and YLD is warranted.

The World Health Organization has already taken some measures to facilitate improvements in patient safety with its Global Patient Safety Challenges. Each of these measures highlighted a particular patient safety issue, which led to the development of front-line interventions through a partnership with member states, with the aim for this to be implemented in each country. In 2005, “Clean Care is Safer Care” was launched, focusing on hand hygiene improvement [25]. A few years later, in 2008, WHO launched “Safe Surgery Saves Lives” to reduce risks associated with surgery [26]. The most recent challenge was “medication without harm,” launched in 2017. The aim is to reduce the level of severe, avoidable harm related to medications by 50% globally. The three areas covered in this launch are the high-risk medications, polypharmacy, and transitions of care [27]. Such initiatives to reduce AEMT are essential. However, formal evaluations of the impact and outcome of these programs have been challenging to undertake [28]. These strategies may bring patient safety and avoidance of AEMT to the forefront globally. However, to tackle the increasing trend observed in YLD, a more robust approach together with the formal evaluation of existing strategies and data sharing across various stakeholders is required.

Our study has several implications for global health care delivery. Although the number of people dying because of AEMT has decreased over the years, little has been done to reduce the morbidity component of AEMT. Our findings question the effectiveness of health care delivery programs and policies to reduce the global burden of AEMTs related to disability. Hence, a revised bundle of public health measures, including preventive clinical safety education, newer appropriate electronic medical record documentation, and decision support is needed. Hopefully, this will enable the scientific community to accurately pinpoint the amount and causes of the occurrence of AEMTs, thereby reducing the long-term morbidity of AEMT.

Our study highlights potential issues with traditional health care planning. The process is outcome-driven with a historical emphasis on reducing mortality as its main aim [29]. Ongoing advances in the provision of medical care have resulted in a reduction in mortality rates. This, however, has the unintended but understandable consequence of a significant number of individuals living longer with chronic disability, thus explaining the global increase in YLD. There is also the risk that a reduction in DALY will be used as a measure for assessing cost-effectiveness for essential services like rehabilitation. This could theoretically make recovery difficult and, in turn, keep individuals locked in a state of chronic disability [30].

The limitations of this study include reliance on secondary data, which depends on the quality and completeness of the primary sources. Since AEMT-associated disabilities are often underreported, these estimates should be interpreted with caution. Regions with higher burdens of severe diseases and shortages of health care professionals may be prone to higher AEMT rates, which may not be reported or recorded for a variety of reasons. On the other hand, health systems with robust mechanisms for non-punitive disclosure of medical errors may report more incidents, leading to reporting bias. The primary aim of our study was to quantify the variation and trends across the WHO regions relating to DALY. However, we also reported a few of the selected countries based upon them having the highest population within their respective regions. We acknowledge the heterogeneity of health care resources and practices across different countries. Although the findings of our study are more applicable to a review of the DALY across the WHO regions, these may not be applicable to generalize conclusions across different countries. Instead, detailed research may be required to identify the variations between different countries more accurately. Finally, we predicted the future decrease in DALY based on previous trends. These findings may not provide a reliable basis for predicting the future DALY burden because of unexpected circumstances such as natural catastrophes and changes in economic developments of the regions and countries. Nevertheless, such trends provide a possible direction for policymakers to provide strategies to reduce the burden related to AEMT.

## Conclusions

In the present study, we observed an overall decline in DALY rates due to AEMT between 1990 and 2017. The DALY rates differed by age and sex and among WHO regions, where low income and economically developing countries contribute more weight to the global DALY burden. Worldwide, we found an increase in YLD suggesting an increased burden of people living with poor health due to AEMT. The forecasting analyses showed a gradual decrease in DALY until 2040 across WHO regions. Our study highlights the importance of global collaborations for education, research, and the engagement of the general public and health care professionals to decrease the global and regional burden of AEMT.
